# ER Morphology in the Pathogenesis of Hereditary Spastic Paraplegia

**DOI:** 10.3390/cells10112870

**Published:** 2021-10-25

**Authors:** Sonia Sonda, Diana Pendin, Andrea Daga

**Affiliations:** 1 Department of Biomedical Sciences, University of Padua, 35121 Padua, Italy; sonia.sonda@phd.unipd.it; 2 Neuroscience Institute, Italian National Research Council, 35121 Padua, Italy; 3 Laboratory of Molecular Biology, Scientific Institute IRCCS E. Medea, 23842 Bosisio Parini, Italy

**Keywords:** endoplasmic reticulum, hereditary spastic paraplegia, ER-shaping proteins

## Abstract

The endoplasmic reticulum (ER) is the most abundant and widespread organelle in cells. Its peculiar membrane architecture, formed by an intricate network of tubules and cisternae, is critical to its multifaceted function. Regulation of ER morphology is coordinated by a few ER-specific membrane proteins and is thought to be particularly important in neurons, where organized ER membranes are found even in the most distant neurite terminals. Mutation of ER-shaping proteins has been implicated in the neurodegenerative disease hereditary spastic paraplegia (HSP). In this review we discuss the involvement of these proteins in the pathogenesis of HSP, focusing on the experimental evidence linking their molecular function to disease onset. Although the precise biochemical activity of some ER-related HSP proteins has been elucidated, the pathological mechanism underlying ER-linked HSP is still undetermined and needs to be further investigated.

## 1. Clinical Features of HSP

HSP comprises a group of clinically and genetically heterogeneous neurodegenerative disorders caused by a broad range of mutations affecting the so-called SPG genetic loci. HSP is rare, showing an overall estimated prevalence of about 1–10/100,000 [1]. However, its distribution is highly variable among different regions (also due to discrepancies in the classification and diagnosis). HSPs share as a common feature a bilateral spasticity and weakness of the lower-extremities, as emerging by neurological examination [2]. The severity of these symptoms varies among patients, and they are often accompanied by lower-extremity hyperreflexia and extensor plantar responses.

The HSPs are progressive disorders whose hallmark is the degeneration of the cortical motor neurons that project axons to the spinal cord. Loss or damage of axons is evident by radiological examination in the corticospinal tract, although they are not exclusively confined to this region. Indeed, the corpus callosum, frontal and temporal lobes, cerebellum, as well as other brain regions display imaging changes [3], indicating the involvement of a broad range of neuronal subtypes. Although there is an indication that radiological defects correlate with severity and progression of the disease, the association with the genotype of the patients is still weak [3].

Clinically, HSPs have been classified as “pure” (or uncomplicated) HSPs when symptoms are limited to lower limb weakness and spasticity, likely arising from degeneration of the corticospinal tract, or as “complicated” HSPs when patients present other symptoms such as cerebellar ataxia, peripheral neuropathy, optic atrophy, seizures, and dementia. Complicated symptoms likely derive from dysfunctions of additional brain regions and neuronal types. Although these symptoms often correlate with the specific genotype of the patient, they usually complicate the diagnosis due to overlap with signs and symptoms of other neurodegenerative diseases (e.g., ataxias, dementias, amyotrophic lateral sclerosis) [4]. This may also suggest that HSPs are not a single disease but a group of different diseases whose common feature is degeneration of the corticospinal tracts.

Symptom onset is also highly variable, ranging from early childhood (classified as “early onset”) to later age. Early onset HSP are most often characterized by non-progressive symptoms, while later onset HSPs usually worsen slowly but unceasingly. Although HSPs are disabling, the life span of patients is not shortened. No cure is available to prevent or reverse nerve degeneration in HSP and current treatments simply target symptom reduction [3].

## 2. Genetics of HSP

The genetic diversity of HSP is considerable, with more than 80 HSP genes defined by genetic linkage analysis. The relative frequency of each HSP gene mutation varies substantially by geographic region. The three HSP genes most commonly found mutated in patients are Spastin, Atlastin-1, and REEP1 (Receptor expression-enhancing protein 1), accounting for about 50% of total cases, while other HSP genes have been found mutated in only a few consanguineous families. Although many HSP genes have been identified, a large set of them remains unknown [5].

Almost all types of inheritance have been documented for HSP, the most common being autosomal dominant (AD, 75–80% of affected individuals). Autosomal recessive (AR, 25–30%), X-linked and mitochondrial inheritance (1–2% collectively) have been reported with lower frequencies. 

**Figure 1:**
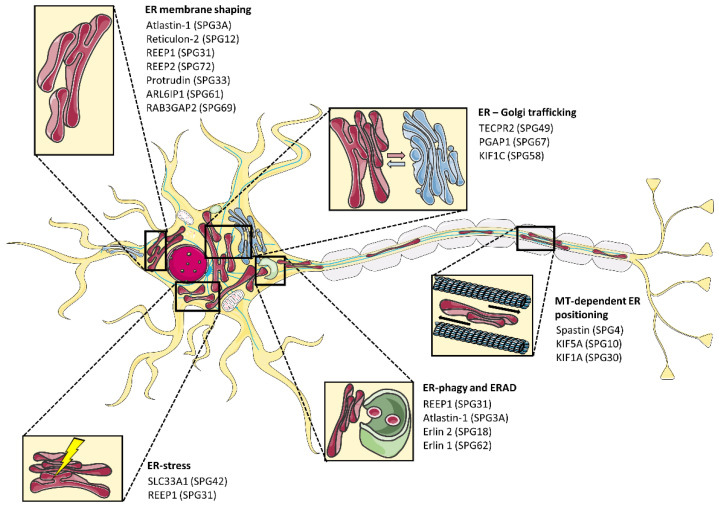
HSP proteins involved in ER-related functions fundamental for neuronal maintenance.

SPG proteins have been implicated in a number of cellular pathways, such as ER shaping, axonal transport, membrane trafficking, protein folding and ER-stress, lipid metabolism, and axon myelination [6,7]. Remarkably, around half of AD-HSP patients carry mutations affecting proteins that have a direct role in ER shaping (e.g., Atlastin-1, Reticulon-2, or REEP1) or act on cellular pathways that indirectly have an impact on ER tubules shape or positioning (e.g., cytoskeleton maintenance, ER-phagy, ER stress) (Figure 1 and Table 1).

Strikingly, if one considers only pure HSP (i.e., those characterized by clinical symptoms likely arising from degeneration of the corticospinal tract), the vast majority of cases are due to mutations in genes involved in the maintenance of ER shape/function [6,8]. This suggests a tight relationship between ER shape/function and corticospinal axon maintenance, although the reason why only subpopulations of neurons are affected by the potential morphological changes caused by mutation of ER-shaping proteins is still unknown.

## 3. ER Shaping Proteins

The ER is a continuous membrane network that extends throughout the cytoplasm of eukaryotic cells. The peripheral ER comprises a network of tubules and sheet-like structures in continuity with the nuclear envelope. Traditionally, ER cisternae have been classified as ribosome bound, “rough” ER (RER), while ER tubules are considered ribosome-free, “smooth” ER (SER). The purpose of differently shaped ER domains has not been fully established. Cisternae are characterized by a larger luminal volume vs. surface area, compared to tubules, suggesting that they would be the preferred site for processes, such as protein folding, taking place in the ER lumen [23,24,25]. Conversely, ER tubules are considered the preferential location for lipid synthesis and membrane protein accumulation [24]. Accordingly, the relative abundance of RER and SER in different cell types often correlates with their function (with secretory cells, for example, containing mainly RER) [26]. Nevertheless, recent evidence suggests that the distinction between ER tubules and cisternae can be blurred. Structural analysis performed on EM sections showed that the transformation of long cisternae into tubules during mitosis in cultured cells occurs via the progressive formation and expansion of fenestrations [25]. Moreover, it has been reported that ER tubules can also be decorated with ribosomes, albeit at lower densities than in cisternae [24,25]. Thanks to super-resolution imaging technologies, other “unconventional” ER structures have been discovered that have traditionally been categorized as cisternae due to the limited spatiotemporal resolution of standard optical microscopy. These comprise clusters of highly dense peripheral tubules [27] as well as sheets fenestrated by “nanoholes” [28] and ribosome-associated vesicles [29]. 

Owing to its particular shape and function, the neuronal ER needs further tweaks and adjustments to fit in cell bodies, and especially in dendrites or axons. Indeed, neuronal cell bodies contain both SER and RER with highly dynamic transitions between each other [30]. Stacked sheets are connected by helicoidal structures, providing an extended surface for ribosomes [31] to perform protein translation. The axonal ER is primarily tubular, with an enrichment in a population of very narrow tubules (~20–30 nm in diameter) [32]. The RER constitutes a very small fraction of axonal ER [33], suggesting a limited contribution of ER-associated ribosomes to local protein translation. The narrow tubules within axons and dendrites do not connect together in three-way junctions as in cell bodies, probably due to curvature constraints as a result of the small diameter of these neuronal protrusions. Instead, they connect at the edges of sheets with high curvature [32]. 

Altogether, these findings support a model for ER network based on a highly dynamic continuum of membrane structures that can undergo rapid interconversion in order to support fast rearrangements of the peripheral ER. Although the purpose of the constant reorganization of the ER network is not completely understood, it is reasonable to speculate that ER dynamics help reconfigure the spatial organization of the network in response to specific intracellular stimuli. A few basic processes contribute to ER dynamics, including fusion and fission, elongation, retraction, and branching of tubules. In recent years, progress has been made in understanding how the architecture of the ER is generated and maintained, thanks in particular to the discovery of ER-shaping proteins that are specifically designed to create and maintain the highly complex structure of the ER [34].

### 3.1. Atlastin-1 (SPG3A)

Human Atlastin-1 (ATL1) is a GTPase that, on the basis of its structural features, can be classified as a member of the dynamin family of large GTPases and was first identified as the protein encoded by *SPG3A*, the gene found mutated in the earliest onset form of AD-HSP [9]. Two additional proteins extensively related to Atlastin-1 were later discovered in the human genome and thus named Atlastin-2 (ATL2) and Atlastin-3 (ATL3). ATL3 has also been implicated in human disease as its mutation is responsible for hereditary sensory and autonomic neuropathy (HSAN) [35,36]. Mammalian atlastins differ in tissue expression, with ATL1 being mainly expressed in the central nervous system (CNS) [9,37] whereas ATL2 and ATL3 are more ubiquitously expressed [38]. These proteins share two transmembrane helices in addition to the GTPase domain, suggesting that they all reside within cellular membranes [37]. Although the structural fingerprint of ATL1 unequivocally reveals an ability to hydrolyze GTP, the functional role of this GTPase activity within the cell remained unknown for some time. An early subcellular localization was misleading: ATL1 was shown to be a Golgi-resident protein [39], possibly with a very limited localization within ER membranes. This interpretation led to the initial hypothesis that ATL1 might be involved in ER–Golgi membrane trafficking [39], perhaps in membrane fusion events due to its topological similarities to the mitofusins, or in vesicle budding and fission because of its resemblance to dynamin. Interestingly, it was later established that ATL2 and ATL3 localized specifically to the ER [38]. The extraordinary homology among human atlastins, however, suggested that all three proteins would be targeted to the same membranes. A key confirmation of the actual subcellular localization of atlastins came from *Drosophila* where a single atlastin ortholog was identified. Indeed, endogenous *Drosophila* atlastin was found in vivo to localize primarily to the ER [40,41] and definitive ER localization was finally determined also for ATL1, when it was shown to interact physically with the ER-specific protein Reticulon-4a and to play a role in ER shaping [42]. Nevertheless, the actual function of ATL1 remained unresolved since only a broad hypothesis was proposed, based largely on the analogy to other members of the dynamin family of GTPases, that ATL1 would promote either the fusion or fission of ER tubules [42]. The existence of one atlastin gene in *Drosophila* helped elucidate its precise cellular function. In vivo approaches combined with the in vitro proof that fly atlastin is sufficient to catalyze lipid bilayer fusion when incorporated into synthetic lipid vesicles demonstrated that atlastin is the mediator of the homotypic fusion of ER membranes [40]. Although Drosophila atlastin remains to date the only atlastin, with the exception of the very distantly related yeast [43] and plant orthologs [44], capable of promoting membrane fusion in vitro, it is accepted by extension that also vertebrate atlastins possess this functional property in vivo.

Atlastins consist of a GTPase (G) domain, a helix bundle (HB) domain, two closely spaced transmembrane segments (TM1 and TM2), and a *C*-terminal tail (CT) containing an amphipathic helix (AH) [45,46,47]. Structural studies of the cytosolic domains of ATL1 have produced some confounding results that have predictably led to conflicting models for the mechanism of atlastin-mediated membrane fusion [46,47]. A plausible model that reconciles the available crystal structures begins with GTP binding to atlastin monomers. In the next step, the monomers form a dimer with the bound GTP molecules buried at the interface. GTP hydrolysis by the dimer drives the association of the two HBs, forming a tight dimer. Finally, Pi and GDP are released sequentially, causing the dissociation of the dimer into monomers. The sequence of reactions leading to dimerization can occur between atlastin molecules that reside in the same or different membranes (cis- or trans- interactions, respectively), but membrane fusion can only occur during trans-interactions.

A large number of mutations rather uniformly distributed across all domains of the ATL1 protein have been identified as the cause of SPG3A disease. These mutations include small deletions, small insertions, splice site mutations, and whole exon deletions. However, the large majority is represented by missense variants. A review of the literature indicates that there is no clear genotype-phenotype correlation in HSP-SPG3A patients [48]. Since SPG3A is a dominant disease, it might be expected that all disease variants would have a crippling impact on the function of ATL1. However, the membrane fusion activity of ATL1 cannot be tested directly since human atlastins do not possess the ability to catalyze the fusion of synthetic membranes in vitro. Furthermore, experiments that address ATL1 fusion in vivo or in cell culture have not produced satisfactory functional answers for reasons that clearly include genetic redundancy due to the existence in vertebrates of three highly homologous atlastin genes that most probably serve overlapping functions. To overcome this limitation, the fusogenic activity of Atlastin-1/SPG3A pathological mutations has been assessed by reproducing point mutations in the Drosophila protein and testing the mutant fly atlastin in vitro. However, only a few of the more than 70 pathogenic mutations identified to date have been analyzed in this manner [46], in part since mutations that do not occur in conserved aminoacids cannot be replicated. Another strategy to evaluate the impact of ATL1 pathological variants has employed analysis in the soluble phase to test their ability to hydrolyze GTP and dimerize in a nucleotide-dependent manner as a proxy for functional performance but, for many of the variants examined, the impairment of GTP hydrolysis and dimer formation turned out to be rather modest [46,47]. Consequently, neither the studies on *Drosophila* atlastin nor those probing the biochemical properties of ATL1 were able to uncover a clear correlation between SPG3A disease-causing mutations and protein activity. This lack of understanding of the pathological process is further underscored by the results of investigations relying on the properties of *Drosophila* atlastin in cell culture, in vitro and in vivo. Exogenous *Drosophila* atlastin was shown to be able to functionally replace human atlastins in HeLa cells depleted of the endogenous proteins [49], but simultaneous assessment of conserved pathological mutations in the *Drosophila* orthologue on both ER morphogenesis using this replacement assay and membrane fusion catalysis in vitro did not provide a better understanding of the genotype-phenotype correlation for SPG3A [50]. Likewise, the analysis of a few atlastin CRISPRed knock-in pathological mutant fly lines indicated that no obvious correlation can be identified between the behavior of pathological mutations in vivo and their activity [50,51,52]. The work by Montagna et al. has helped to partially elucidate the pathogenetic mechanism of atlastin mutations, although the results should not be generalized because only four mutants were analyzed. While the prevailing theory posits that most atlastin mutations act through a dominant-negative mechanism [53,54], this work showed that in vivo in flies both overexpression of these four pathological variants and heterozygosity of the same variants in CRISPRed individuals, do not elicit the loss-of-function effects expected for dominant-negative mutations, suggesting instead that the variants tested share a straightforward loss-of-function mechanism [52].

How is ER membrane fusion linked to HSP? This remains one crucial, unanswered question. From the biochemical standpoint, atlastin is a transmembrane enzyme that uses GTP hydrolysis to fuel the fusion of ER membranes. No other activities have been associated with this protein. Consequently, it can be assumed that pathological mutation of ATL1 would interfere with the establishment and/or maintenance of proper ER morphology through perturbation of membrane fusion. Nevertheless, the available data have only provided circumstantial experimental support in favor of this hypothesis. Although this may be due in part to the lack of suitable animal models, the main reason may be more related to the challenges associated with imaging the complex structure of the neuronal ER in vivo to determine morphological differences between wild-type and mutant ER, which are still insurmountable. The uncertainty regarding the consequences induced by ATL1 mutation in the neuronal ER, has led to questioning the idea that SPG3A mutations cause HSP exclusively by disrupting membrane fusion and ER morphogenesis [50].

An alternative possibility is that atlastins are indeed directly involved only in membrane fusion but indirectly implicated in other ER-related cellular processes whose disruption eventually leads to disease even when ER shape is not visibly affected. The ER is responsible for protein synthesis, modification and quality control, and plays crucial roles in carbohydrate metabolism, control of lipid synthesis and delivery, formation of other membrane-bound organelles, and lipid droplets as well as Ca^2+^ homeostasis [55,56]. Fusion impairment below the current threshold of detection, such as that probably caused by dominant mutation of ATL1, could then affect, in ways that we still do not understand, one or several ER functions. ATL2 GTPase activity has been reported to contribute to IP3-induced dendritic Ca^2+^ signals in primary hippocampal neurons [57] and depletion of ATLs affects store-operated Ca^2+^ entry in PC-12 cells [58], hinting that atlastins may influence Ca^2+^ homeostasis. This notion may be supported by a report that Atlastin-1 modulates seizure activity and neuronal excitability [59] and the observation that *Drosophila* atlastin decreases evoked transmitter release at the neuromuscular junction and causes age-dependent decline in adult locomotion [57,60], even though both works surmise that interference with BMP trafficking could also be a potential source of these phenotypes, conceivably suggesting that perturbation of trafficking could underlie HSP-disease. Indeed, HSP-causing mutations were shown to disrupt BMP receptors II trafficking to the cell surface in HEK239-T cells [61] and knockdown of zebrafish atl1 decreases larval mobility, perturbs the architecture of spinal motor axons and is associated with a substantial upregulation of the BMP signaling pathway, again implying that atlastins may regulate BMP receptor trafficking [62]. Another possible mechanism of SPG3A disease, linked but secondary to membrane fusion, is the regulation of lipid droplet size. In fact, gain- and loss-of-function studies in *C. elegans*, *Drosophila*, and mammalian cells have suggested that the atlastins affect LD size, most likely in an indirect manner, through their ER membrane fusion activity [63]. Additionally, all atlastins have been implicated in ER-phagy where their role requires a functional GTPase domain and proper ER localization [64] and ATL3 functions specifically as a receptor for GABARAP during ER-phagy, with HSAN-associated ATL3 mutations disrupting association with GABARAP and impairing ATL3 function in ER-phagy [65].

In conclusion, while several different pathways could lead secondarily to atlastin-associated disease, the ability to catalyze membrane fusion is the only function demonstrated for atlastin(s) to date and evidence-based mechanistic links between fusion and other proposed disease pathways leading to HSP have not been corroborated making defects in membrane fusion the most likely route to SPG3A disease.

### 3.2. Reticulon-2 (SPG12)

Reticulons (RTNs) comprise a large family of conserved membrane-spanning proteins residing in the ER. RTNs apparently arose early in the evolution of eukaryotes, where they are ubiquitous, although the number of genes encoding these proteins varies widely among species (4 RTN genes in vertebrates (RTN1-4), 2 in yeast (RTN1 and RTN2), 1 in *Drosophila melanogaster*, 1 in *Caenorhabditis elegans*, 21 and 17 in *Arabidopsis thaliana* and *Oryza sativa* plants, respectively [66,67,68]). Mutations in RTN2 have been linked to human disease and shown to be the cause of SPG12, an autosomal dominant form of pure HSP [10].

RTNs contain a so-called ‘reticulon homology domain’ (RHD), a sequence of 150–200 amino acid residues located at the *C*-terminus characterized by two unusually long hydrophobic regions interrupted by a hydrophilic loop, which is of great importance for the localization and function of the protein [66]. The RHD is the only structural feature common to all RTNs, whose *N*-terminal part, instead, is extremely variable both in length, which ranges from a few to over one thousand amino acids, and in sequence. The hydrophobic stretches of the RHD have been demonstrated to insert into lipid bilayers as a hairpin [69] that, working like a wedge, occupies more space on the outer leaflet of the lipid bilayer than on the inner leaflet, thereby producing positive membrane curvature. Through this mechanism, RTNs have been shown to stabilize curved membrane tubules and sheet edges contributing to shape the complex architecture of this organelle [70]. Moreover, in vivo and in vitro analyses using the *Drosophila* RTN homolog (Reticulon-like-1, Rtnl1), have shown that this protein, in addition to generating the static curvature of the moderately curved membranes of ER tubules and sheet edges, produces also extreme curvature and constriction of dynamic tubules leading to their fission, thus acting as a counterbalance to atlastin-mediated fusion in the regulation of ER morphology [71].

Since atlastins and RTNs are involved in the maintenance of ER shape, it is reasonable to speculate that disruption of this process through pathological mutation would ultimately underpin their role in the pathogenesis of HSP. While for ATL1 there is some evidence converging on this interpretation, support for a similar conclusion about RTNs is still scarce. The main obstacles to understanding how RTNs might cause HSP disease are, first, the difficulty in sorting out the phenotypes associated with loss of RTNs function and, second, their putative general involvement in neurodegeneration. RTNs have indeed been implicated in several neurodegenerative diseases such as Alzheimer’s, Parkinson’s, amyotrophic lateral sclerosis, and multiple sclerosis [72,73], although a mechanistic understanding of a possible causal link between the neurodegenerative process and RTNs function is still lacking.

The four mammalian RTNs exist in a number of differentially spliced isoforms that share varying degrees of homology and have potentially redundant roles that are presently not fully understood. Indeed, knock-out mice for RTN1, RTN3, and RTN4 have been generated but all homozygous mutant individuals are viable and seemingly normal, potentially suggesting a compensatory mechanism among the RTNs. In addition, examination of the ER by electron microscopy shows that loss of the RTNs in different types of cells of these mice had no effect on its tubular structure [74,75,76,77,78]. This is consistent with a report showing that even the simultaneous loss of RTN1, RTN3, and RTN4 in U2O2 cells only partially converts tubules to sheets [79] as well as with another work where in a double-deletion mutant of the two RTN genes in *S. cerevisiae* the morphology of the peripheral ER network was normal [69]. In these models, the contradictory effects of the loss of RTNs on ER morphology seem to indicate that their individual role in shaping this organelle is dispensable, suggesting instead that a concerted action may be required. However, their collective role also remains vague. First, because the loss of multiple RTNs in cells and yeast has consequences that are not always consistent with their putative function [69,79], and second because the differential expression of the four RTNs in different tissues and cell types (i.e., RTN1 and RTN3 expression is essentially limited to the nervous system [72,77,78] and RTN4 is expressed ubiquitously [66,80]) complicates our understanding of their various functional combinations. These experimental observations make it difficult to envisage why so many different RTN isoforms with overlapping functions should have evolved, cooperating in different combinations to eventually achieve the identical result of generating the membrane curvature typical of ER tubules and sheet edges.

Despite these difficulties, recourse to simpler model systems that lack the redundancy of vertebrates has partially helped to unravel the function of RTNs. Loss of *Drosophila* Rtnl1 was shown to cause most of the tubular ER to be converted to unbranched sheets, even though, as in mice, it didn’t impact the survival of flies [33,71]. Rtnl1 role in shaping the ER was further confirmed by the demonstration that it engages in a functionally antagonistic interaction with the fusion protein atlastin by mediating membrane fission [71]. However, these findings raise an interesting issue: if ER morphology is linked to function, and RTNs play an important role in controlling ER shape, then why do flies with an ER obviously altered by the absence of their single Rtnl1 survive unscathed? One possibility is that ER shape is not so crucial under normal circumstances and becomes critical only under specific conditions that have not yet been identified. The enrichment in ER sheets observed in flies and, to some extent, in cell lines following loss of RTNs [71,79] poses another problem as RTNs are thought to stabilize not only the high curvature of tubules but also that of sheet edges which are abundant in cells and tissues lacking RTNs due to the conversion of tubules to sheets. Other families of sequence-unrelated RHD-containing proteins, such as REEPs/DP1/Yop1p, reside in the ER and have been grouped together with RTNs as being responsible for the generation of ER curvature [70,81,82] and thus could be implicated in compensating for the missing RTNs. Nevertheless, in vivo data are essentially absent and in vitro evidence is available only for sparse members of these families and thus does not permit to prove the widely accepted paradigm that they are functionally equivalent in shaping the ER network. Furthermore, it seems highly unlikely that proteins belonging to different families were independently selected during the course of evolution to interchangeably perform an identical cellular function. Finally, we note that a possible compensatory effect of REEP/DP1/Yop1p would have to be specifically targeted to sheet edges, which are abundant upon loss of RTNs, without causing a similar compensation in the membrane of the tubules. However, this is mechanistically inexplicable based on the mainstream hypothesis that both families of curvature-stabilizing proteins are indiscriminately responsible for creating the curvature of sheet edges and tubules [70,82,83]. It is clear that our comprehension of the function/s of RTNs is rather imperfect, and the inconsistencies presented here clearly demonstrate the need to critically revise current, sometimes contradictory, ideas about ER curvature-inducing proteins and devise experimental strategies to illuminate their individual roles while refraining from extending functional findings for any of these proteins, even for evolutionarily very distant homologs, to members of other families based on assumptions rather than experimental evidence.

In addition to the complexities associated with the role of RTNs on ER shape, our understanding of RTN-dependent HSP is further complicated by the fact that RTNs have been, somewhat generically, implicated in controlling or participating in a number of cellular processes whose impairment may lead to the development of pathological conditions associated with CNS degeneration [73]. Although RTN1 and RTN4 are the isoforms predominantly linked to neurodegeneration, our knowledge remains very limited and it could be speculated that HSP-causing RTN2 mutations could also lead to disease through one of the still unknown pathogenetic pathways to neurodegeneration secondary to perturbation of ER morphology. Unfortunately, direct experimental data on RTN2 are truly scant and its specific function as well as its role in HSP pathogenesis remain elusive.

### 3.3. REEP1 (SPG31) and REEP2 (SPG72)

Mutations in *REEP1* give rise to the third most common dominant form of HSP, accounting for 6.5% of cases [84], while *REEP2* mutations are rare. The most common mutations described for REEP1 are missense mutations, insertion, or deletions that cause premature stop codons [85]. The REEP family of proteins was first identified for their role in trafficking odorant receptors [86] and G-protein coupled receptors to the plasma membrane [87]. They are part of REEPs/DP1/Yop1p family of proteins, which shares a RHD (see above) believed to be responsible for their association with ER membranes. The yeast homolog Yop1p is one of the best characterized members of the superfamily. Yop1p reconstituted into liposomes has been shown to generate tubules [70,88], leading to the hypothesis that this family of proteins is responsible for ER membrane tubulation.

Six mammalian REEPs (REEP1-6) have been identified. However, while REEP5-6 displays the highest homology with Yop1p, REEP1-4 is characterized by the presence of a shorter first hydrophobic segment and the lack of a *N*-terminal cytoplasmic domain [89], likely resulting in the absence of the first transmembrane domain [88]. REEP1 and REEP2 also possess a *C*-terminal tubulin binding domain, which has not been identified in REEP5-6 [89]. These structural divergences are found also in lower-complexity organisms. *Caenorhabditis elegans*, *Strongylocentrotus purpuratus* (sea urchin) and *Drosophila melanogaster* have two REEP orthologs, one with high similarity to REEP1-4 and one with greater similarity to REEP5-6 [89], supporting the notion that REEP1-4 proteins may have functions partially distinct from those performed by REEP5-6/Yop1p.

Structural studies on Yop1p indicated that the transmembrane domains in the RHD contain enough helical residues to fully traverse the hydrophobic bilayer of the ER. In addition, a *C*-terminal amphipathic helix (APH) was identified, whose deletion abolishes membrane tubule formation in vitro [83,88]. The APH region is widely conserved among REEP1-6 and is likely shared also by RTNs [83,88], suggesting a conserved role in membrane shaping. Nevertheless, tubule formation has been reported in vitro upon reconstitution into liposomes only for Yop1p [70,88]. Curiously, *Xenopus laevis* REEP4 and REEP5 homologs or *Drosophila melanogaster* REEP5-6 homolog ReepB, have been reported to form tubules in vitro only in the presence of the atlastin yeast homolog Sey1p and GTP [83,90]. Moreover, Yop1p has been shown to generate extreme curvature at high concentrations by converting phospholipid bilayers into micelle-like lipoprotein particles (LPPs) [83], while *Xenopus* REEP5 generates LLPs (but never tubules) even at very low concentrations. Unfortunately, in vivo evidence corroborating these in vitro observations is sorely missing. Overall, this suggests that although the transmembrane and APH domains of REEP proteins have been broadly associated with ER membrane-shaping properties, the precise role of individual REEP family members in the formation and maintenance of the ER is still poorly understood. 

Similarly, the functional difference between REEP1-4 and REEP5-6 is still obscure. The expression levels of REEP1-2 are low, compared to REEP5-6, although they are enriched in mammalian neuronal tissues [91,92]. Indeed, EGFP fusions at the C-termini of all possible isoforms of the *Drosophila* homolog of REEP1-4 (ReepA) or REEP5-6 (ReepB) revealed a weak expression of ReepA, which was barely detectable in the fly central nervous system and undetectable in the axonal or presynaptic ER. In agreement with this, the loss of ReepB caused ER sheet expansion and partial loss of ER in *Drosophila* distal motor axons, while these effects are essentially undetectable after ReepA knock-out [93]. Surprisingly, it was found that simultaneous loss of mammalian REEP3 and REEP4 in HeLa cells causes ER sheets expansion at the expenses of tubules exclusively during cell mitosis, although they do not seem to be required for the maintenance of ER morphology during interphase [94]. 

This slew of scattered data complicates our understanding of the role of REEP1 and REEP2 in the pathogenesis of HSP. Although some studies have reported ER shaping defects in cells or animal models where REEP1-2 function has been abolished [14,89,95], conclusive evidence that the REEPs function in biogenesis and maintenance of the ER network and that this is the principal pathogenetic route is lacking and will require further investigation.

## 4. Microtubule (MT) Dynamics

MTs are dynamic, polarized cytoskeletal components implicated in a wide range of cellular processes, including cargo transport and organelle positioning. Thanks to their highly dynamic structure, MTs undergo cycles of rapid growth and disassembly in a process defined as dynamic instability [96]. This provides the rapid reorganization of the cytoskeleton necessary for many cellular functions, such as cell division and migration and formation of cell polarity. Experiments in *Xenopus* egg extracts and proteoliposomes have shown that the cytoskeleton is not necessary for the formation of the ER tubular network [97] and, consistently, recent evidence showed that a minimal set of ER-shaping proteins when reconstituted into liposomes are sufficient to form an ER network de novo [90]. Nevertheless, MTs have been shown to co-align with ER tubules [98] and several studies reported a role for the cytoskeleton in the maintenance of ER network. Notably, the role of MTs in ER tubules movement is well-established. In particular, three distinct mechanisms of MT-based ER tubule dynamics have been identified: (i) tip attachment complex (TAC); (ii) ER sliding [99,100]; and (iii) depolymerization of MT ends (dTAC) [98]. Each of these mechanisms can lead to tubule extension; when tubules intersect, they can fuse and create the three-way junctions that compose the polygonal ER network [100,101]. Furthermore, the extension of ER tubules by association with growing MT ends allows the organelle to populate the entire volume of the cell, as well as the length of axons and dendrites [102]. 

Although the cytoskeleton contributes to ER dynamics, it is not clear whether it is required for the maintenance of a pre-existing ER network. Several observations suggest that in mammalian cells, MT cytoskeleton dynamics influence ER tubule distribution and sheet/tubule balance [99,100,103,104], e.g., depolymerization of MTs by nocodazole in cultured mammalian cells inhibits new tubule growth and causes retraction of ER tubules from the cell periphery [98,105]. The reliance of ER tubule movement on MT dynamics suggests that mutation of proteins acting on MTs may have an effect on ER structure. Indeed, several proteins involved in MT dynamics (e.g., Spastin, KIF1A, KIF5A) have been found mutated in HSP. The relationship between cytoskeletal control of ER dynamics and diseases has been recently reviewed [106].

### Spastin (SPG4)

About 40% of pure AD-HSP are caused by mutations in the *SPG4/SPAST* gene, encoding Spastin [15]. Spastin, a member of the AAA (ATPase associated with various cellular activities) family, is a MT-severing protein that plays a major role in cytoskeleton regulation [107,108,109,110]. In addition to the ATPase domain, it also contains a MT Binding Domain (MTBD) necessary for MT binding, and a MT Interacting and Transport (MIT) domain [107]. To exert its severing activity, Spastin is believed to assemble into hexamers, dock on MTs and break them up [111,112]. Recently, it has been discovered that severases can also promote MT growth likely through a mechanism in which the MT fragments created by severing act as seeds for the growth of new MTs [113,114]. Moreover, Spastin severing activity is regulated by MT post-translational modification [115,116,117]. Considering these features altogether, it is clear that fine control of Spastin function is necessary for regulating MT number, mobility and distribution in the cell [108,109,110,118]. 

Over 300 mutations and deletions have been described in the SPG4 gene. The wide spectrum of mutations includes non-sense, frameshift, splice site, missense mutations, as well as extended deletions. Loss-of-function (haploinsufficiency or dominant-negative) has been proposed as the pathological mechanism [110,119,120,121], supported by the fact that mutant Spastin proteins have not been detected in cells and tissues derived from human patients [121,122], as well as by experiments in a *Drosophila* SPG4 model [123]. However, two independently generated Spastin knockout mouse models failed to recapitulate major HSP hallmarks [124,125], questioning the validity of the haploinsufficiency model. On this basis, the contribution of an additional gain of function mechanism has been proposed for specific missense mutations [126].

The *SPAST* gene produces a 530 amino acid (60 kDa) ubiquitous isoform called M87 and a much less abundant 616 amino acid (68 kDa) isoform called M1 whose expression is restricted to the spinal cord [127]. The MIT, MTBD and AAA domains are present in both M1 and M87 Spastin isoforms, whereas M1 Spastin features a specific 86-aminoacid *N*-terminal domain containing a hydrophobic region suggested to form a hairpin that can partially insert into the ER membrane. Spastin has been reported to interact with ER resident proteins ATL1, RTN1 and REEP1 via its hydrophobic hairpin [89,128,129,130,131]. Despite suggestions that these interactions may be important for ER-MT interplay [89,107,129,132], their actual role in the building and maintenance of the tubular ER network is still unclear.

The precise role of Spastin at the ER remains unknown, since no requirement for endogenous Spastin in ER shaping has yet been demonstrated. Available evidence shows that ER morphology is affected when Spastin is downregulated or when pathogenic variants are expressed [131,133,134]. Experiments in *Drosophila* showed that the expression of the pathological variant spastin^K467R^ (known to induce MT hyper-stabilization via a dominant-negative effect) shifts the ER sheets/tubules balance toward the formation of sheets. However, administration the MT-destabilizing drug vinblastine, which has been shown to restore MT organization [123], rescues also ER morphology, suggesting that ER perturbation occurs as a consequence of the impairment of MT dynamics [134]. Thus, currently available evidence does not support a direct role for Spastin in regulating ER network shape. Likewise, it has been suggested that Spastin regulates ER-endosome tethering, lysosome size, and LD size [135,136,137,138], but there is no conclusive proof that these effects are associated with activities of the protein beyond its MT-destabilizing role. In summary, the specific biochemical function of Spastin and the lack of evidence that it exerts additional primary functions suggest that disruption of MT dynamics is the most likely pathological mechanism underlying SPG4, although the consequences may interfere with multiple MT-dependent cellular processes.

## 5. ER-Golgi Trafficking

Proper intracellular trafficking is essential for neuronal development and function [139] and neurons appear particularly sensitive to defects in trafficking [140]. Indeed, early secretory compartments ER, ER-exit sites (ERES) and ER-Golgi intermediate compartment (ERGIC) are distributed throughout the soma, dendrites and axon, while the Golgi apparatus and Golgi outposts are found only in the somatodendritic compartment and are excluded from axons [141]. Thus the trafficking to specific neuronal domains must be finely regulated for proper neuronal function and dysregulation of this process may contribute to neurological disease pathogenesis. Mutations in genes involved in ER-Golgi trafficking have also been associated with HSP [139,140,142].

Anterograde ER-to-Golgi transport requires COPII-coated vesicles that form at ERES [143]. ERES-generated COPII vesicles are directed towards ERGIC, from where cargo proteins can be sorted to the Golgi apparatus, retained in the ERGIC or sent back to the ER [144]. In addition to ERES, COPII vesicles can bud from ER-phagy sites (ERPHS) and then be sorted to autophagosomes [145]. 

Several ERES constituents act as regulators for subunit recruitment and complex stabilization [144]. Among these interactors, the TRK-fused gene (TFG, *SPG57*) protein [19,146] and the Tectonin Beta-Propeller Repeat Containing 2 (TECPR2, OMIM *SPG49*) protein [18] have been associated with complicated forms of HSP when mutated. TFG interacts directly with the scaffolding protein Sec16 [147] and ER morphology and distribution were found altered in TFG-depleted COS-7 cells [19]. TECPR2 is a Atg8-binding partner for autophagy [18,148] thought to be required for COPII-dependent cargo export from ER to Golgi [140,149]. HSP patient fibroblasts carrying mutant TECPR2 showed delayed ER export [149]. Consistently, TECPR2 depletion in mammalian cells reduced ERES number [149] and led to the expansion of ER sheets, suggesting a link between ER-Golgi transport and maintenance of ER morphology [149]. TECPR2 depletion has been shown to block the delivery to the Golgi apparatus of glycosylphosphatydilinositol (GPI)-anchored proteins (GPI-APs) [149,150]. GPI deacylation is crucial for efficient transport of GPI-APs from the ER to the Golgi [151]. Intriguingly, SPG67 is caused by an autosomal recessive mutation in the gene coding for the post-GPI attachment to proteins inositol Deacylase 1 (PGAP1) [13], an ER membrane protein required for GPI-APs deacylation. 

Pathological mutations engineered in the endogenous *Drosophila* atlastin gene impacted Golgi structure in neurons [52], and transfection of mutant ATL1 in HEK293 cells prevented Golgi maturation [152]. A reduction in the efficiency of cargo packaging into COPII vesicles and delayed cargo delivery to Golgi has been observed upon ATLs depletion/mutation both in mammalian [153,154] and plant cells [155], potentially underscoring the importance of the connection between ER shape and function.

In addition to the classical COPI-coated vesicle-dependent retrograde Golgi to ER transport [154], a COPI-independent pathway has been described [154,156,157]. In particular, cytoskeleton-based mechanisms are suggested to participate in this process through the repositioning of Golgi membranes [158]. KIF1C (*SPG58*) is a MT-binding protein belonging to kinesin superfamily that has been found mutated in HSP [159]. KIF1C participates in Golgi-to-ER trafficking [160] and appears to function in the maintenance of Golgi structure, since its depletion has been reported to cause Golgi fragmentation in HeLa cells [159,161]. Two other kinesins have been found mutated in HSP, KIF5A (*SPG10*) [16,162,163] and KIF1A (*SPG30*) [17,164], but they are involved in axonal organelle transport [165,166], possibly affecting ER positioning rather than ER-to-Golgi transport.

## 6. Other Proteins Implicated in ER Morphogenesis and Function

Other SPG proteins have been proposed to localize to ER membranes and play a role in ER shaping. Although the precise cellular function of these proteins is not completely clear, the possibility that other HSP proteins, directly or indirectly, may affect ER morphogenesis further supports the idea that ER maintenance is essential for axonal wellness.

ARL6IP1 (ADP-ribosylation factor-like 6 interacting protein 1, *SPG61*) contains short hairpin transmembrane domains and its overexpression induced ER tubulation with exclusion luminal protein in mammalian cells. For this reason, ARL6IP1 has been proposed to be involved in shaping ER tubules in a Reticulon-like fashion [167]. However, depletion of the protein in *Drosophila* causes ER fragmentation [168] thus questioning the validity of the proposed hypothesis.

Rab3GAP2 (Rab3 GTPase-activating protein non-catalytic subunit, *SPG69*) is a component of Rab3GAP complex required for the recruitment and activation of Rab18 to the ER membrane [13]. Depletion of Rab3GAP2 resulted in loss of Rab18 at the ER and reorganization of ER sheets, which extend to the cell periphery [169]. The precise mechanism underlying this modification, however, remains unexplained.

The ER coordinates a variety of cellular processes fundamental for the life of the cell, including protein synthesis, folding and modification, translocation of secretory proteins, synthesis of phospholipids and steroids, storage of calcium. Known ER shaping proteins as well other HSP-causing proteins not directly implicated in the regulation of ER morphology, have been reported to affect additional ER-related processes, such as ER-phagy and ER stress response [33,170,171,172,173]. However, there is no evidence showing a direct role for ER-shaping proteins in the ER stress response that is likely a secondary consequence of morphology alteration.

## 7. Conclusions

Despite the genetic heterogeneity of HSPs, alterations in morphology and/or distribution of the ER appear to be a critical causative factor. This places ER at the nexus of HSP pathology, suggesting that ER morphology/function plays an important role in neuronal degeneration associated to HSP. Neurons are particularly reliant on an elaborate ER network for proper functionality since their unique architecture requires the peripheral ER to propagate throughout both axons and dendrites. The relationship between the structure and function of ER is only beginning to be explored, and the link between defects in ER morphology/function and the onset of HSPs is under intense investigation but remains largely unexplained.

## Figures and Tables

**Table 1:** HSP genes with established (or proposed) role in the maintenance of ER shape/function (MT, microtubule; P, pure; C, complex).

Main Pathway	SPG	Gene	Protein	Inheritance	Frequency	Pure/Complex	Molecular Function Relevant to ER	Reference
ER membrane shaping	SPG3A	ATL1	Atlastin-1	AD	Second most frequent AD form (≈10%)	P (C)	ER membrane fusion	[9]
	SPG12	RTN2	Reticulon-2	AD	Rare	P	ER membrane tubulation and fission	[10]
	SPG31	REEP1	Receptor expression-enhancing protein 1	AD	3–9% of all AD HSP	P (C)	ER tubular network organization	[11]
	SPG33	ZFYVE27	Protrudin	AD	Rare (1 family)	P	ER network distribution	[12]
	SPG61	ARL6IP1	ADP-ribosylation factor-like protein 6-interacting protein 1	AR	Rare (1 family)	C	ER tubular network organization	[13]
	SPG69	RAB3GAP2	Rab3 GTPase-activating protein non-catalytic subunit	AR	Rare (1 family)	C	ER network organization	[13]
	SPG72	REEP2	Receptor expression-enhancing protein 2	AD/AR	Rare (2 families)	P	ER tubular network organization	[14]
MT-dependent ER positioning	SPG4	SPAST	Spastin	AD	Most frequent AD form (≈40%)	P (C)	MT severing	[15]
ER-Golgi trafficking	SPG10	KIF5A	Kinesin heavy chain isoform 5A	AD	1–2% of AD HSP	P (C)	MT-dependent transport	[16]
	SPG30	KIF1A	Kinesin-like protein KIF1A	AR	Rare	P or C	MT-dependent transport	[17]
	SPG49	TECPR2	Tectonin beta-propeller repeat-containing protein 2	AR	Rare	C	Anterograde transport	[18]
	SPG57	TFG	Protein TFG	AR	Rare (1 family)	C	Anterograde transport	[19]
	SPG58	KIF1C	Kinesin-like protein KIF1C	AR	Rare	P or C	MT-dependent transport	[20]
	SPG67	PGAP1	GPI inositol-deacylase	AR	Rare (1 family)	C	Anterograde transport	[13]
ER stress	SPG18	ERLIN2	Erlin-2	AR	Rare	C	ERAD	[21]
	SPG42	SLC33A1	Acetyl-coenzyme A transporter 1	AD	Rare (1 family)	P	ER membrane transport	[22]
	SPG62	ERLIN1	Erlin-1	AR	Rare	P	ERAD	[13]
